# MRI analysis of relative tumor enhancement in liver metastases and correlation with immunohistochemical features

**DOI:** 10.1186/s13244-024-01866-7

**Published:** 2024-12-05

**Authors:** Felix Barajas Ordonez, Sebastian Gottschling, Kai Ina Eger, Jan Borggrefe, Dörthe Jechorek, Alexey Surov

**Affiliations:** 1 University Clinic for Radiology and Nuclear Medicine, Otto- von-Guericke-University Magdeburg, Magdeburg, Germany; 2 Department of Diagnostic and Interventional Radiology, University Hospital RWTH, Aachen, Germany; 3 Institute of Pathology, Otto-von-Guericke-University Magdeburg, Magdeburg, Germany; 4 Institute for Radiology, Neuroradiology and Nuclear Medicine, Johannes Wesling University, Ruhr University Bochum, Minden, Germany

**Keywords:** Magnetic resonance imaging, Immunohistochemistry, Breast cancer, Pancreas cancer, Colorectal cancer

## Abstract

**Objective:**

Investigate the association between the relative tumor enhancement (RTE) of gadoxetic acid across various MRI phases and immunohistochemical (IHC) features in patients with liver metastases (LM) from colorectal cancer (CRC), breast cancer (BC), and pancreatic cancer (PC).

**Methods:**

A retrospective analysis was conducted on 68 patients with LM who underwent 1.5-T MRI scans. Non-contrast and contrast-enhanced T1-weighted (T1-w) gradient echo (GRE) sequences were acquired before LM biopsy. RTE values among LM groups were compared by cancer type using analysis of variance. The relationships between RTE and IHC features tumor stroma ratio, cell count, Ki67 proliferation index, and CD45 expression were evaluated using Spearman’s rank correlation coefficients.

**Results:**

Significant differences in RTE were observed across different MRI phases among patients with BCLM, CRCLM, and PCLM: arterial phase (0.75 ± 0.42, 0.37 ± 0.36, and 0.44 ± 0.19), portal venous phase (1.09 ± 0.41, 0.59 ± 0.44, and 0.53 ± 0.24), and venous phase (1.11 ± 0.45, 0.65 ± 0.61, and 0.50 ± 0.20). In CRCLM, RTE inversely correlated with mean Ki67 (*r* = −0.50, *p* = 0.01) in the hepatobiliary phase. Negative correlations between RTE and CD45 expression were found in PCLM and CRCLM in the portal venous phase (*r* = −0.69, *p* = 0.01 and *r* = −0.41, *p* = 0.04) and the venous phase (*r* = −0.65, *p* = 0.01 and *r* = −0.44, *p* = 0.02).

**Conclusion:**

Significant variations in RTE were identified among different types of LM, with correlations between RTE values and IHC markers such as CD45 and Ki67 suggesting that RTE may serve as a non-invasive biomarker for predicting IHC features in LM.

**Critical relevance statement:**

RTE values serve as a predictive biomarker for IHC features in liver metastasis, potentially enhancing non-invasive patient assessment, disease monitoring, and treatment planning.

**Key Points:**

- Few studies link gadoxetic acid-enhanced MRI with immunohistochemistry in LM.
- RTE varies by liver metastasis type and correlates with CD45 and Ki67.
- RTE reflects IHC features in LM, aiding non-invasive assessment.

**Graphical Abstract:**

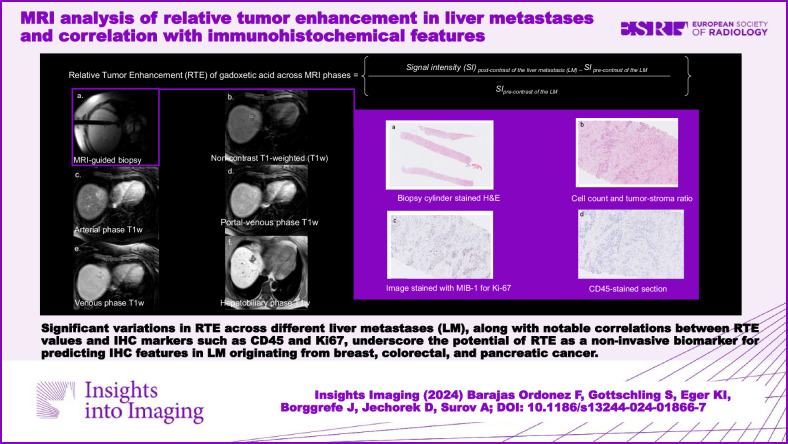

## Introduction

Liver metastases (LM) represent approximately 25% of all secondary lesions in solid organs, frequently originating from colorectal cancer (CRC), pancreatic cancer (PC), and breast cancer (BC) [1]. Among imaging modalities, MRI is widely recognized as the most effective tool for detecting and characterizing LM, achieving high diagnostic accuracy regardless of the primary tumor type [2, 3]. Despite these advances, studies exploring the associations between MRI findings and the cellular and molecular characteristics of intrahepatic malignant lesions, particularly as revealed by immunohistochemistry, remain limited.

Previous studies have begun to explore these associations. Morisaka et al [4] examined the differences in intratumor gadoxetic acid retention across various focal liver lesions during MRI studies. Their study found that the retention index, calculated on pre- and post-contrast T1-weighted (T1-w) images, varied significantly among different types of hepatic malignancies, CRCLM exhibiting lower retention compared to hepatocellular carcinoma (HCC) and intrahepatic cholangiocarcinoma (iCC).

Similarly, Öcal et al [5] further investigated the prognostic value of gadoxetic acid uptake on hepatobiliary phase MRI in 312 patients with HCC who were treated with sorafenib alone or in combination with selective internal radiation therapy. They measured the signal intensity (SI) index of the tumor and normal liver parenchyma on non-contrast and hepatobiliary phase MRI images, defining high gadoxetic acid uptake as relative tumor enhancement (RTE) higher than relative liver enhancement. In their findings, high gadoxetic acid uptake was significantly associated with shorter overall survival (OS). These findings suggest that gadoxetic acid retention may serve as a surrogate parameter for clinical outcomes.

Nonetheless, the correlation between gadoxetic acid-enhanced MRI and immunohistochemical (IHC) features in LM has been investigated to a limited extent. This study aims to investigate the association between RTE of gadoxetic acid across arterial, portal venous, venous, and hepatobiliary MRI phases and its correlation with IHC features such as cell count, Ki-67 proliferation index, tumor stroma ratio (TSR), and CD45 expression in patients with LM from BC, CRC, and PC.

## Materials and methods

### Study design

This was a retrospective study centered on patients with confirmed LM originating from BC, CRC, and PC. The study cohort underwent CT or MRI-guided liver biopsy, before any treatment. A retrospective review of liver biopsies conducted within our department from January 2012 to December 2021 was performed by employing our internal database (MEDICO KIS, CompuGroup Medical SE & Co. KGaA, Koblenz, Germany). The clinical data of the study cohort was retrieved from medical records using our internal database.

**Fig. 1:**
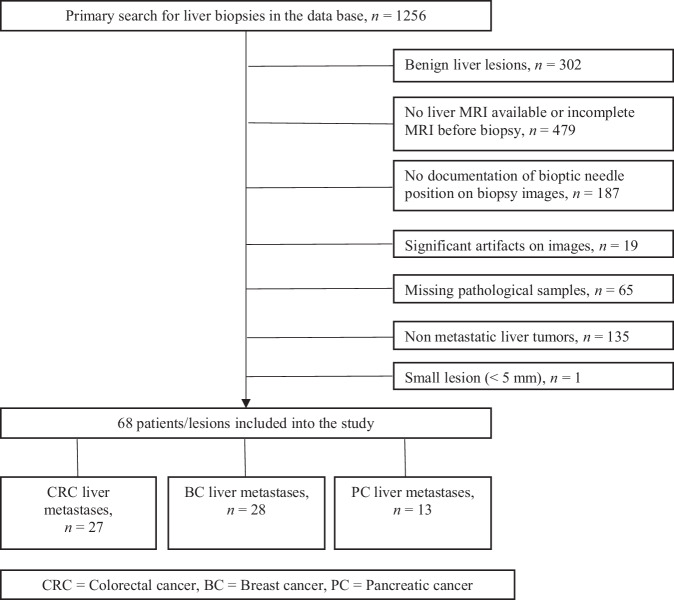
Flow chart of data acquisition

The inclusion criteria comprised: (i) CT or MRI image documentation confirming the biopsy needle position, (ii) a dynamic contrast-enhanced MRI at 1.5 T, conducted within three months before the liver biopsy, and (iii) availability of pathological specimens and IHC information on clinical records. Exclusion criteria encompassed: (i) benign liver lesions, (ii) incomplete MRI sequences or utilization of an MRI contrast agent other than Gd-EOB-DTPA (Primovist®, Bayer Vital, Leverkusen, Germany), (iii) no documentation of biopsy needle position on biopsy images, (iv) significant MRI artifacts, (v) missing pathological samples, (vi) non-metastatic liver tumors, and (vii) LM size < 5 mm (Fig. 1). Liver biopsies were carried out following sterile procedures using 18-G coaxial needles sampling two to three cylindrical specimens in each case.

### MRI technique

**Fig. 2:**
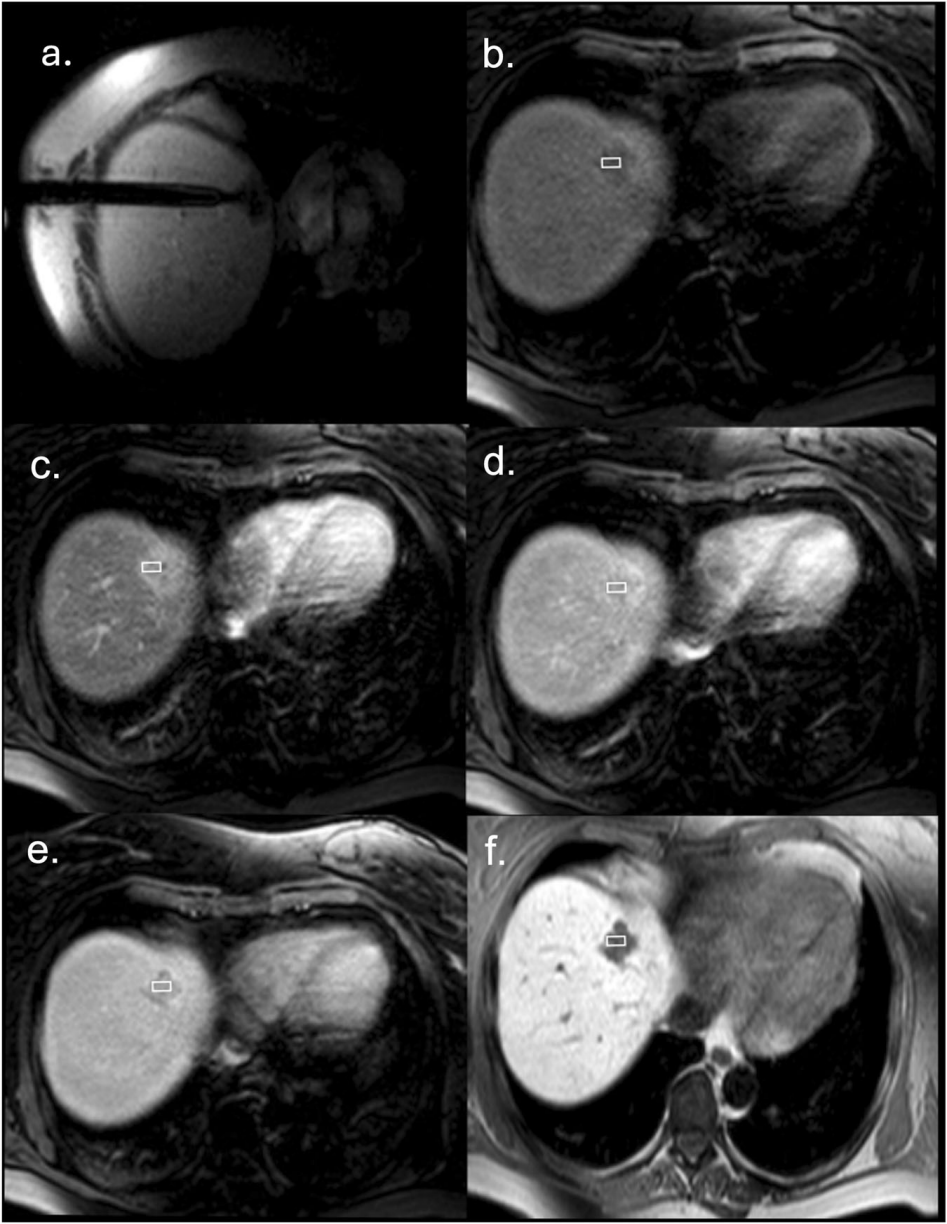
MRI findings of a BCLM. **a** MRI-guided biopsy image showing needle position. A rectangular region of interest is placed within the lesion in each phase according to the needle position on the biopsy image. **b** Non-contrast T1w MRI image. The SI at this stage is measured at 100. **c** Arterial phase T1w MRI image. The SI in this phase is 165, resulting in an RTE of 0.65. **d** Portal-venous phase T1w image. The SI in this phase is 130, with an RTE of 0.30. **e** Venous phase T1w image. The SI in this phase is 125, corresponding to an RTE of 0.25. **f** Hepatobiliary phase T1w image. In this phase, the SI drops to 40, resulting in a negative RTE of −0.60

Each MRI scan was conducted using a 1.5-T MRI scanner, (Achieva, Philips, Best, the Netherlands). The imaging protocol comprised T2-weighted single-shot and turbo-spin echo sequences, both with and without fat suppression. Following the administration MRI contrast agent Gd-EOB-DTPA (0.1 mmol/kg body weight, Primovist®, Bayer Vital, Leverkusen, Germany), dynamic sequences were performed. Non-contrast T1-w gradient echo sequences (GRE) were acquired alongside T1w GRE after contrast administration in the arterial (20–30 s), portal venous (60 s), late venous (3 min), and hepatobiliary phases (20 min) (Fig. 2). The timing of these contrast acquisitions was kept constant throughout the study period.

### Imaging analysis

Image analysis of all patients was conducted independently by two experienced abdominal radiologists (A.S. and S.G.) using a picture archiving and communication system (PACS) viewing station (INFINITT Healthcare, Seoul, South Korea). (i) CT or MRI images confirming the precise position of the biopsy needle within the LM were examined. (ii) Subsequently, the target lesion (TL) was localized within the T1-w GRE sequences. (iii) Using the rectangular function in PACS for manual drawing, rectangular regions of interest (ROIs) were manually drawn in 2D, on non-contrast T1w images closely matching the exact placement of the needle of the CT or MRT-guided biopsy. The ROIs were aligned with the trajectory of the biopsy needle and were confined within the TL while excluding major necrosis areas or peripheral liver parenchyma. The same size ROI was manually replicated across all post-contrast media T1w MRI sequences at the same anatomical level as the biopsy site, ensuring consistency. The ROIs were drawn separately by both radiologists. (iv) Following this, the SI of the TL was measured across non-contrast T1-w GRE sequences using the same slice and localization. (v) The RTE was calculated for each phase with the formula reported by Öcal et al [5]:

$${{\rm{Relative}}}\; {{\rm{tumor}}}\; {{\rm{enhancement}}} = \left\{\frac{({{\rm{SI}}})_{{{\rm{post}}}{\mbox{-}}{{\rm{contrast}}}\; {{\rm{of}}}\; {{\rm{the}}}\; {{\rm{liver}}}\; {{\rm{metastasis}}}\;({{\rm{LM}}})} \; - \; {{\rm{SI}}}_{{{\rm{pre}}}{\mbox{-}}{{\rm{contrast}}}\; {{\rm{of}}}\; {{\rm{the}}}\; {{\rm{LM}}}}}{{{\rm{SI}}}_{{{\rm{pre}}}{\mbox{-}}{{\rm{contrast}}}\; {{\rm{of}}}\; {{\rm{the}}}\; {{\rm{LM}}}}}\right\}$$

### IHC assessment

**Fig. 3:**
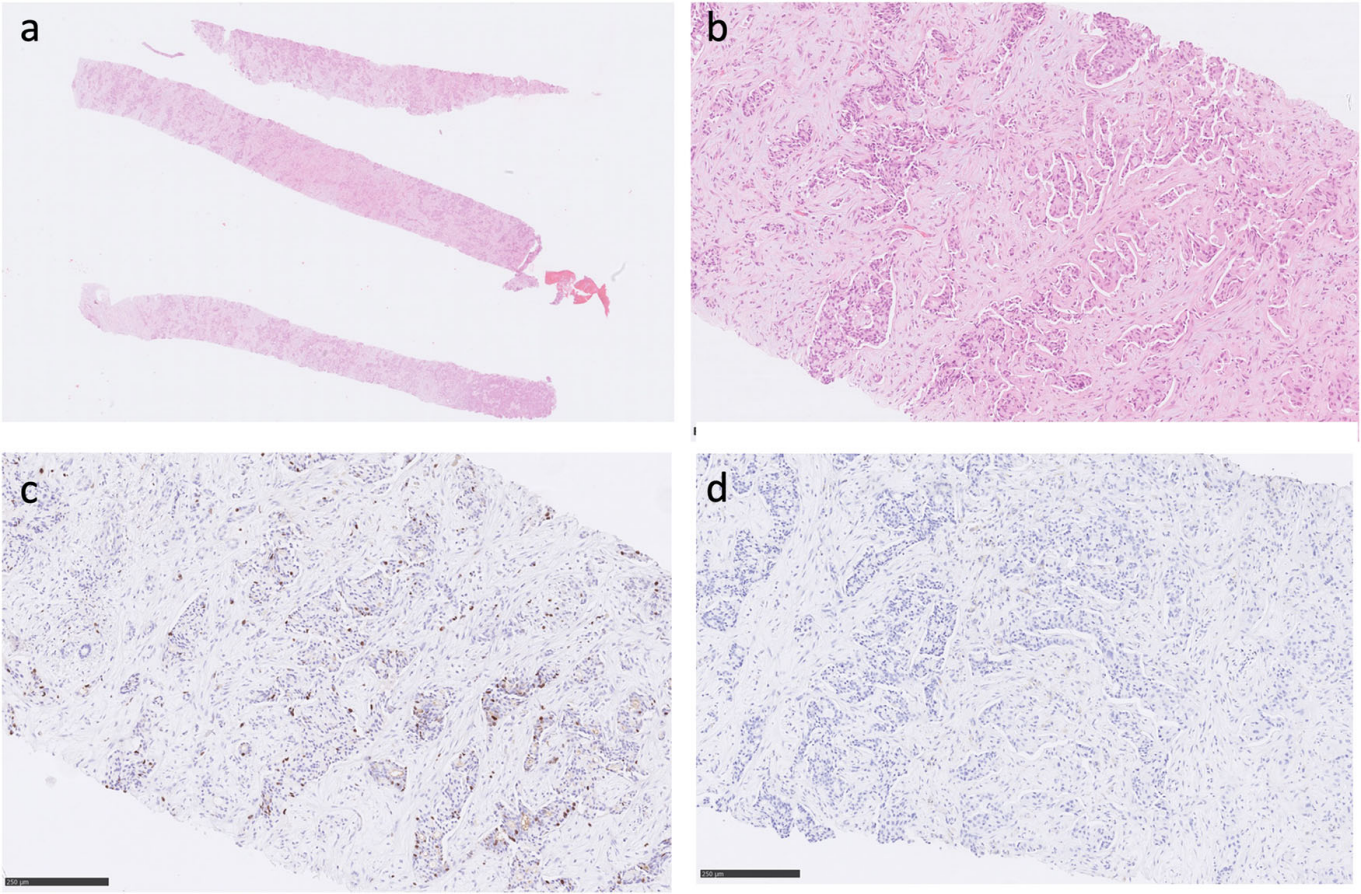
IHC analysis of a BC liver metastasis. **a** Biopsy cylinder stained with H&E illustrating the tissue architecture. **b** H&E staining. The cell count is 166. Tumor-stroma ratio is 15%. **c** Image stained with MIB-1 for Ki-67. The Ki-67 index, indicating proliferative activity, is 40%. **d** CD45-stained section. The mean number of CD45-positive cells is 1

The IHC assessment was conducted by two experienced pathologists, both unaware of the LM imaging details, in a consensus to reach an agreement about the measurements. The IHC analysis was performed retrospectively for all 68 patients, based on previously measured IHC features. Our previously detailed IHC procedure was described in [6] and comprises: (i) formalin-fixed, paraffin-embedded tissues underwent analysis (ii) IHC double staining using hematoxylin and eosin (H&E) was performed. (iii) Antigen detection utilized the Ventana BenchMark ULTRA automated immunohistochemistry slide staining system, iVIEW™ DAB Detection Kit (Roche Diagnostics, Penzberg, Germany), utilizing the indirect biotin-streptavidin method (iv) After staining, counterstaining was performed using Haemalaun solution. (v) Antigen retrieval employed CC1mild, followed by incubation at 36 °C for 32 min with specific primary antibodies recognizing CD45/leukocyte common antigen (polyclonal mouse antibody, clone 2B11 + PD7/26; DAKO/Agilent #M0701) (Dako, Agilent, Santa Clara, USA) or Ki67 (polyclonal mouse antibody, clone Mib1; DAKO/Agilent #M7240) (Dako, Agilent, Santa Clara, USA), diluted to 1:500 or 1:100, respectively. (vi) Mean values for the quantified parameters were computed for each specimen. (vii) TSR assessments were conducted on H&E-stained specimens, presenting percentages separately for tumor and stroma content. (viii) Cell density, including tumor-infiltrating immune cells, was estimated as an average of cell counts or CD45+ leukocytes per high-power field, respectively. (ix) Proliferation rates were determined by the percentage of Ki67-positive cells among all tumor cells (Ki67-index). Each IHC feature underwent evaluation across five power fields (×40 magnification; 0.23 mm^2^ per field) employing the Nikon ECLIPSE Ni-E microscope (Nikon Corporation, Tokyo, Japan) and saved in uncompressed Tagged Image File Format (Fig. 3).

### Statistical analysis

Continuous variables are expressed as mean ± standard deviation (mean ± SD), while categorical variables are presented as counts (n) and percentages (%). To evaluate the interobserver agreement of the RTE values among the LM groups obtained from the independent measurements by two radiologists, the intraclass correlation coefficient (ICC) was used. For this purpose, the RTE was calculated within subsets of ten patients from each individual LM group (CRC, BC, and PC). The interpretation was performed according to Landis and Koch, interpreted as follows: ICC values of 0.61–0.80 indicating substantial and 0.81–1.00 indicating excellent agreement. Differences in mean RTE among the various groups were assessed utilizing analysis of variance. The correlation between RTE and IHC features was assessed using Spearman’s rank correlation coefficient (*r*). A two-tailed *p*-value ≤ 0.05 was considered statistically significant. IBM SPSS Statistics for Windows, version 27.0 (IBM Corp., Armonk, NY, USA) was used for the statistical analysis.

## Results

### Patient characteristics

A total of 68 patients were included in the analysis: 28 with BCLM, 27 with CRCLM, and 13 with PCLM. The BCLM group consisted entirely of female patients (100%), with a mean age of 55.46 ± 6.34 years. In the CRCLM group, the majority were male (81.5%), with a mean age of 63.25 ± 11.58 years. The PCLM group had a more balanced sex distribution (46.2% male) and the highest mean age, 71.13 ± 9.23 years. All patients in the BCLM and PCLM groups were classified as having Stage IV disease, whereas all patients in the CRCLM group were classified as Stage IVA (refer to Suppl. Table 1).

### IHC features

The IHC features of BCLM showed mean values as follows: TSR: 38.37 ± 24.15 (%), cell count 181.86 ± 77.21, CD45 2.80 ± 5.76, and Ki-67 max 26.43 ± 15.95 (%). In the case of CRCLM, the TSR was 43.79 ± 24.23 (%), cell count 182.62 ± 75.74, CD45 1.82 ± 3.10, and Ki-67 max 53.15 ± 27.67 (%). Regarding the PCLM, TSR was 29.05 ± 22.29 (%), the cell count 159.55 ± 57.12, the CD45 4.22 ± 5.08, and the Ki-67 max 33.46 ± 12.14. The IHC features are shown in Table 1.

**Table 1:** Histological features of all LM (*n* = 68)

	Tumor-stroma ratio (%)	Cell count, *n*	CD45, *n*	Ki-67 mean (%)	Ki-67 max (%)
BCLM (*n* = 28)	38.37 ± 24.15	181.86 ± 77.21	2.80 ± 5.76	19.81 ± 13.32	26.43 ± 15.95
CRCLM (*n* = 27)	43.79 ± 24.23	182.62 ± 75.74	1.82 ± 3.10	42.43 ± 26.85	53.15 ± 27.67
PCLM (*n* = 13)	29.05 ± 22.29	159.55 ± 57.12	4.22 ± 5.08	23.46 ± 10.52	33.46 ± 12.14

*LM* liver metastases, *BC* breast cancer, *CRC* colorectal cancer, *PC* pancreatic cancer

### Interobserver agreement of RTE

The interobserver agreement in the RTE for BCLM shows an exceptional agreement, reaching as high as 0.99 in the arterial phase (refer to Suppl. Table 2a). In the case of CRCLM the average agreement across all phases was nearly perfect (refer to Suppl. Table 2b). This pattern in the agreement persisted in RTE of PCLM (refer to Suppl. Table 2c).

### RTE values in the different LM groups

In the arterial phase, the highest RTE values were observed in the BCLM group (0.75 ± 0.42), followed by PCLM (0.44 ± 0.19), and CRCLM (0.37 ± 0.36) (*p* < 0.001) (Table 2). Statistically significant variations in RTE values were also noted across the three groups of LM in the portal venous and venous phases, with BCLM showing 1.09 ± 0.41 and 1.11 ± 0.45, CRCLM showing 0.59 ± 0.44 and 0.65 ± 0.61, and PCLM showing 0.53 ± 0.24 and 0.50 ± 0.20, respectively (*p* < 0.001 for both phases) (Table 2). However, in the hepatobiliary phase, no significant differences were observed among the groups, with RTE values of 0.65 ± 0.69 for BCLM, 0.73 ± 0.74 for CRCLM, and 0.39 ± 0.69 for PCLM (*p* = 0.37) (Table 2).

**Table 2:** RTE across different liver metastasis groups (*n* = 68)

	BCLM	CRCLM	PCLM	*p*-value
Arterial phase	0.75 ± 0.42	0.37 ± 0.36	0.44 ± 0.19	< 0.001
Portal venous phase	1.09 ± 0.41	0.59 ± 0.44	0.53 ± 0.24	< 0.001
Venous phase	1.11 ± 0.45	0.65 ± 0.61	0.50 ± 0.20	< 0.001
Hepatobiliary phase	0.65 ± 0.69	0.73 ± 0.74	0.39 ± 0.69	0.37

*LM* liver metastases, *BC* breast cancer, *CRC* colorectal cancer, *PC* pancreatic cancer

### Correlation analysis of RTE values and IHC features

BCLM Group (Table 3a): RTE exhibited a moderate positive correlation (*r* = 0.41, *p* = 0.03) with CD45 in the portal venous phase. No other significant correlations were identified in this group.

**Table 3:** Correlation analysis between different features

	TSR	CD45	Cell count	Ki67 mean	Ki67 max
(a)					
Arterial phase	*r* = −0.27 (*p* = 0.17)	*r* = 0.21 (*p* = 0.27)	*r* = 0.10 (*p* = 0.6)	*r* = 0.13 (*p* = 0.51)	*r* = 0.12 (*p* = 0.53)
Portal venous phase	*r* = −0.16 (*p* = 0.42)	*r* = 0.41 (*p* = 0.03)	*r* = 0.19 (*p* = 0.34)	*r* = 0.08 (*p* = 0.70)	*r* = 0.02 (*p* = 0.94)
Venous phase	*r* = 0.11 (*p* = 0.58)	*r* = 0.14 (*p* = 0.37)	*r* = −0.18 (*p* = 0.37)	*r* = −0.08 (*p* = 0.68)	*r* = −0.08 (*p* = 0.70)
Hepatobiliary phase	*r* = 0.06 (*p* = 0.76)	*r* = −0.13 (*p* = 0.51)	*r* = −0.22 (*p* = 0.26)	*r* = −0.18 (*p* = 0.35)	*r* = −0.17 (*p* = 0.38)
(b)					
Arterial phase	*r* = 0.17 (*p* = 0.39)	*r* = −0.31 (*p* = 0.11)	*r* = − 0.07 (*p* = 0.74)	*r* = 0.04 (*p* = 0.85)	*r* = 0.06 (*p* = 0.78)
Portal venous phase	*r* = −0.01 (*p* = 0.98)	*r* = −0.41 (*p* = 0.04)	*r* = 0.09 (*p* = 0.67)	*r* = −0.05 (*p* = 0.82)	*r* = −0.02 (*p* = 0.93)
Venous phase	*r* = −0.05 (*p* = 0.79)	*r* = −0.44 (*p* = 0.02)	*r* = 0.06 (*p* = 0.02)	*r* = −0.20 (*p* = 0.32)	*r* = −0.19 (*p* = 0.35)
Hepatobiliary phase	*r* = 0.14 (*p* = 0.47)	*r* = −0.10 (*p* = 0.62)	*r* = −0.30 (*p* = 0.12)	*r* = −0.50 (*p* = 0.01)	*r* = −0.55 (*p* = < 0.01)
(c)					
Arterial phase	*r* = −0.57 (*p* = 0.03)	*r* = 0.01 (*p* = 0.98)	*r* = −0.06 (*p* = 0.84)	*r* = 0.10 (*p* = 0.72)	*r* = 0.06 (*p* = 0.84)
Portal venous phase	*r* = −0.35 (*p* = 0.22)	*r* = −0.69 (*p* = 0.01)	*r* = 0.21 (*p* = 0.47)	*r* = 0.42 (*p* = 0.14)	*r* = 0.47 (*p* = 0.09)
Venous phase	*r* = −0.19 (*p* = 0.52)	*r* = −0.65 (*p* = 0.01)	*r* = −0.01 (*p* = 0.97)	*r* = 0.40 (*p* = 0.16)	*r* = 0.47 (*p* = 0.09)
Hepatobiliary phase	*r* = 0.04 (*p* = 0.89)	*r* = −0.16 (*p* = 0.58)	*r* = 0.04 (*p* = 0.89)	*r* = −0.31 (*p* = 0.28)	*r* = −0.14 (*p* = 0.62)

*TSR* tumor stroma ratio

(a): Correlation analysis between the RTE and IHC features in BCLM (*n* = 28)

(b): Correlation analysis between the RTE and IHC features in CRCLM (*n* = 27)

(c): Correlation analysis between the RTE and IHC features in PCLM (*n* = 13)

CRCLM Group (Table 3b): RTE showed moderate negative correlations with CD45 in both the portal venous (*r* = −0.41, *p* = 0.04) and venous (*r* = −0.44, *p* = 0.02) phases. A moderate negative correlation was identified between RTE and Ki-67 mean in the hepatobiliary phase (*r* = −0.50, *p* = 0.01) and between RTE and Ki67 max in the same phase (*r* = −0.55, *p* < 0.01). Regarding cell count, a positive weak correlation was observed solely in this group in the venous phase (*r* = 0.06, *p* = 0.02).

PCLM Group (Table 3c): a moderate negative correlation was found between TSR and RTE in the arterial phase (*r* = −0.57, *p* = 0.03). Additionally, significant negative correlations were observed between RTE and CD45 expression in both the portal venous (*r* = −0.69, *p* = 0.01) and venous phases (*r* = −0.65, *p* = 0.01).

## Discussion

Our study investigated the association between RTE of gadoxetic acid across various MRI phases—arterial, portal venous, venous, and hepatobiliary phases—and key IHC features in LM originating from CRC, BC, and PC. Significant variations in RTE were observed among different LM types, with correlations between RTE and specific IHC features such as Ki-67 proliferation index, CD45 expression, TSR, and cell count differing by LM type and MRI phase.

In the arterial phase, RTE values significantly differed among the LM groups, with BCLM showing the highest RTE, followed by PCLM and CRCLM. The BCLM group continued to show the highest RTE values in the portal venous and venous phases, indicating a potentially  distinct vascular profile compared to CRCLM and PCLM. Our findings align with those of Morisaka et al [4], who demonstrated significant variations in gadoxetic acid retention across different hepatic malignancies, with CRCLM generally exhibiting lower uptake compared to other tumor types. Noda et al [7] demonstrated that different contrast uptake patterns in PCLM were associated with varying OS rates, and Öcal et al [5] reported that a high RTE in HCC was correlated with shorter OS in patients compared to those with lower RTE. In contrast to Öcal et al [5], who used circular ROIs drawn on MRI images at the tumor's largest diameter, we employed rectangular ROIs aligned with the CT- or MRI-guided biopsy needle placement and trajectory. This approach ensured that the imaging analysis corresponded directly to the biopsy sites, leading to accurate correlations between imaging and IHC features. Our findings suggest the need for further investigation to determine whether the observed differences in RTE among LM groups could serve as surrogate markers for clinical outcomes or the identification of LM from known primary tumors. In the hepatobiliary phase, the absence of significant differences in RTE values among the LM groups (*p* = 0.37) suggests that hepatobiliary uptake is less influenced by the vascular characteristics observed in earlier phases.

Gadoxetic acid-enhanced MRI has been proven valuable in predicting tumor behavior, particularly in HCC. Studies, such as those performed by Kitao et al [8] and Fujita [9], suggest that lower SI is indicative of higher proliferative activity in HCC. Kitao et al [8] found significant negative correlations between SI and serum biomarkers like AFP, AFP-L3, and PIVKA-II, which are associated with more advanced histological malignancy and worse overall prognosis. In a more recent study, Yang et al [10] demonstrated that microvascular invasion in HCC could be effectively predicted using gadoxetic acid-enhanced MRI. Their study found that lower gadoxetic acid uptake in the hepatobiliary phase was significantly associated with higher rates of microvascular invasion, which is a critical factor in tumor aggressiveness and patient prognosis. Similarly, in our study, moderate negative correlations were identified between RTE and Ki-67 mean in the hepatobiliary phase (*r* = −0.50, *p* = 0.01) and Ki-67 max (*r* = −0.55, *p* < 0.01) in the CRCLM group, suggesting that lower RTE could indicate higher proliferative activity and more aggressive tumor behavior. This may also suggest that similar relationships between RTE and tumor aggressiveness in HCC may also apply to CRCLM.

Moreover, in the CRCLM group, we found an inverse relationship between RTE and CD45 in the venous and portal venous phases (*r* = −0.41, *p* = 0.04 and *r* = −0.44, *p* = 0.02), suggesting that reduced gadoxetic acid uptake might indicate a more active immune microenvironment. Since CD45 is associated with immune cell activity and various cellular processes [11], this finding supports the hypothesis that reduced gadoxetic acid uptake may reflect a more immunologically active tumor microenvironment [10]. Kemper et al [12] reported in their study of liver biopsies from patients with primary esophageal adenocarcinoma, higher densities of CD45-positive cells were associated with significantly longer OS, establishing CD45 as an independent prognostic marker. While our results suggest that MRI could non-invasively reflect aspects of tumor immune activity, further research is needed to directly correlate these imaging biomarkers with immune infiltration and validate these findings through histopathological data.

The relationship between MRI parameters and IHC features has also been established using apparent diffusion coefficient (ADC) when measured by diffusion-weighted imaging. Karaman et al (2015) [13] observed a negative correlation between ADC and Ki-67 in non-small cell lung cancer. Besa et al (2016) [14] investigated the use of ADC for characterizing histopathologic tumor grade in neuroendocrine tumor LM. They identified a negative correlation between ADC and tumor grade and between ADC and Ki-67. Further studies have also explored the relationship between ADC and tumor biology, Meng et al [15] identified a correlation between ADC values and cell proliferation. In our study, we identified an inverse correlation between RTE and Ki67 mean (*r* = −0.50, *p* = 0.01) and Ki67 max (*r* = −0.55, *p* < 0.01) in the hepatobiliary contrast phase in CRCLM. These findings suggest that RTE could serve as a surrogate marker for assessing tumor aggressiveness and differentiation in CRCLM, similar to the role of ADC in other tumor types. Finally, for the cell count, a weak positive correlation was noted solely in the CRCLM group in the venous phase (*r* = 0.06, *p* = 0.02), with no significant correlations observed in the other types of LM across the different MRI phases. This suggests that the relationship between RTE and cell count may be more nuanced and possibly influenced by other factors that were not fully explored in this study.

In the PCLM group, a moderate negative correlation between TSR and RTE in the arterial phase (*r* = −0.57, *p* = 0.03) suggests that tumors with higher stromal content may exhibit reduced arterial phase enhancement. The prevalence of high TSR has been demonstrated to vary across different malignancies, influencing tumor behavior [16]. However, variability in imaging characteristics associated with TSR has not yet been thoroughly established. Unlike the studies by Xi et al [17] and Pyo et al [16], which highlighted the prognostic significance of TSR across various cancers, further research is needed to determine whether this correlation between RTE and TSR in PCLM reflects clinical outcomes. Additionally, significant correlations between CD45 expression and RTE were observed in the portal venous (*r* = −0.69, *p* = 0.01) and venous phases (*r* = −0.65, *p* = 0.01), similar to the CRCLM group, suggesting that lower RTE in these contrast phases is associated with higher immune cell infiltration.

Conversely, in the BCLM group, a positive correlation was found between CD45 expression and RTE in the portal venous phase (*r* = 0.41, *p* = 0.03), indicating a different pattern of immune response compared to CRCLM and PCLM. This finding suggests that in BCLM, higher immune cell infiltration may correspond with increased RTE in the portal venous phase, potentially reflecting a unique interaction between the tumor and its immune microenvironment. The distinct pattern observed in BCLM aligns with the understanding that various tumor types interact differently with their microenvironments, influencing both their biological behavior and imaging characteristics [18]. Additionally, BC is grouped into four subtypes based on molecular characteristics and IHC features, exhibiting greater molecular heterogeneity compared to other tumors [18]. In contrast, CRC and PC are often driven by more consistent genetic mutations, such as KRAS mutations, present in approximately 40% of CRC and 90% of PC cases, respectively [19, 20]. Given the small sample size of BCLM (*n* = 28) in our study, a detailed subanalysis of different RTE patterns in the BC subtypes remains a topic for further investigation.

In conclusion, RTE values vary across different LM depending on the primary tumor type. RTE may serve as a non-invasive biomarker for predicting distinct IHC features in LM from CRC, BC, and PC. Specifically, RTE serves as a potential imaging surrogate parameter for intratumoral CD45 expression in BCLM in the portal venous contrast phase and in CRCLM and PCLM in both portal venous and venous contrast phases. Furthermore, in the hepatobiliary contrast phase, RTE negatively correlates with aggressiveness as indicated by Ki67 mean and Ki67max values in CRCLM. Although the underlying mechanisms remain incompletely understood, our findings suggest that RTE could significantly impact treatment strategies and disease progression monitoring in patients with LM.

There are several limitations to this study that should  be acknowledged. First, the small sample size for each of the three analyzed LM types (CRC, BC, and PC), and the exclusion of a wider range of LM from other tumor entities, may limit the generalizability of our findings. Additionally, assessing IHC correlations among LM originating from different primary tumors relies on consistent staining techniques and interpretation methods, which may vary across different centers. Variability in any of these parameters can impact the accuracy of correlations observed. Another limitation of our study is the use of 2D ROIs placed on a single slice, which may not fully capture the heterogeneity of some LM and may underestimate tumor enhancement in regions outside the ROI. While this method was chosen to align the imaging analysis with the exact biopsy site and avoid spatial mismatches, it has limitations in assessing the full extent of lesion variability. Furthermore, the information regarding previous treatments was not consistently documented across the different departments that referred patients for biopsies in radiology; therefore, this information was not included in the analysis. In future studies exploring each metastasis separately, the role of previous therapies should be determined. While this exploratory study provides insights into the correlations between RTE of gadoxetic acid MRI and IHC findings, a larger sample size from multiple centers would enhance the precision and power of these correlations, and its clinical relevance should be determined. Therefore, future multicentric studies with larger cohorts are warranted to validate and strengthen the findings of this study.

## Supplementary information

ELECTRONIC SUPPLEMENTARY MATERIAL

## Data Availability

All data generated or analyzed during this study are included in this article and its online supplementary material files. Further information can be provided by the corresponding author upon reasonable request.

## Abbreviations

ADC: Apparent diffusion coefficient
BC: Breast cancer
CRC: Colorectal cancer
GRE: Gradient echo
HCC: Hepatocellular carcinoma
IHC: Immunohistochemical
LM: Liver metastases
OS: Overall survival
PACS: Picture archiving and communication system
PC: Pancreatic cancer
ROI: Region of interest
RTE: Relative tumor enhancement
SI: Signal intensity
TL: Target lesion
TSR: Tumor stroma ratio
