# Supplementary material for: MRI analysis of relative tumor enhancement in liver metastases and correlation with immunohistochemical features
Source: Insights Imaging. 2024 Dec 5;15:294. doi: 10.1186/s13244-024-01866-7 (PMC11621246; doi:10.1186/s13244-024-01866-7)
Supplement: Supplementary file 1 — ELECTRONIC SUPPLEMENTARY MATERIAL [file 13244_2024_1866_MOESM1_ESM.pdf]

MRI Analysis of Relative Tumor Enhancement in Liver Metastases and Correlation with Immunohistochemical Features

ELECTRONIC SUPPLEMENTARY MATERIAL

Suppl. Table 1a. Patient characteristics (*n* = 68)

LM group	Number of patients (n)	Sex (Male/Female) (n, %)	Age (Mean ± SD years)	Tumor Stage (n, %)
BCLM	28	28/0 (0%, 100%)	55.46 ± 6.34	Stage IV 28 (100 %)
CRCLM	27	22/5 (81.50%, 18.50%)	63.25 ± 11.58	Stage IVA 27 (100 %)
PCLM	13	6/7 (46.20%,53.80%)	71.13 ± 9.23	Stage IV 13 (100 %)

SD, standard deviation; LM, liver metastases; BC, breast cancer; CRC, colorectal cancer; PC, pancreatic cancer.

Suppl. Table 2a. Inter-observer agreement of RTE for breast cancer liver metastases

Phase	Inter-observer agreement	*p*-value
Arterial	0.998	< 0.001
Portal venous	0.978	< 0.001
Venous	0.806	0.007
Hepatobiliary	0.997	< 0.001

RTE, relative tumor enhancement

Suppl. Table 2b. Inter-observer agreement of RTE for colorectal liver metastases

Phase	Inter-observer agreement	p-value
Arterial	0.989	< 0.001
Portal venous	0.992	< 0.001
Venous	0.980	< 0.001
Hepatobiliary	0.995	< 0.001

RTE, relative tumor enhancement

Suppl. Table 2c. Inter-observer agreement of RTE for pancreatic cancer liver metastases

Phase	Inter-observer agreement	p-value
Arterial	0.977	< 0.001
Portal venous	0.996	< 0.001
Venous	0.992	< 0.001
Hepatobiliary	0.971	< 0.001

RTE, relative tumor enhancement
